# Ebola Virus Glycoprotein IgG Seroprevalence in Community Previously Affected by Ebola, Sierra Leone

**DOI:** 10.3201/eid2803.211496

**Published:** 2022-03

**Authors:** Daniela Manno, Philip Ayieko, David Ishola, Muhammed O. Afolabi, Baimba Rogers, Frank Baiden, Alimamy Serry-Bangura, Osman M. Bah, Brian Köhn, Ibrahim Swaray, Kwabena Owusu-Kyei, Godfrey T. Otieno, Dickens Kowuor, Daniel Tindanbil, Elizabeth Smout, Cynthia Robinson, Babajide Keshinro, Julie Foster, Katherine Gallagher, Brett Lowe, Macaya Douoguih, Bailah Leigh, Brian Greenwood, Deborah Watson-Jones

**Affiliations:** London School of Hygiene and Tropical Medicine, London, UK (D. Manno, P. Ayieko, D. Ishola, M.O. Afolabi, F. Baiden, B. Köhn, K. Owusu-Kyei, G.T. Otieno, D. Kowuor, D. Tindanbil, E. Smout, J. Foster, K. Gallagher, B. Lowe, B. Greenwood, D. Watson-Jones);; Mwanza Intervention Trials Unit, National Institute for Medical Research, Mwanza, Tanzania (P. Ayieko, D. Watson-Jones);; University of Sierra Leone College of Medicine and Allied Health Sciences, Freetown, Sierra Leone (B. Rogers, A. Serry-Bangura, O.M. Bah, I. Swaray, B. Leigh);; Janssen Vaccines and Prevention, Leiden, the Netherlands (C. Robinson, B. Keshinro, M. Douoguih)

**Keywords:** Ebola virus, viruses, zoonoses, IgG, antibodies, seroprevalence, risk factors, Sierra Leone, Africa

## Abstract

We explored the association of Ebola virus antibody seropositivity and concentration with potential risk factors for infection. Among 1,282 adults and children from a community affected by the 2014–2016 Ebola outbreak in Sierra Leone, 8% were seropositive for virus antibodies but never experienced disease symptoms. Antibody concentration increased with age.

Ebola virus (EBOV) antibodies have been found in populations that have never experienced documented Ebola outbreaks and in persons who reported no history of Ebola virus disease (EVD) (*1*). The clinical significance of these findings is unknown. We conducted a cross-sectional study in healthy adults and children from a population affected by the 2014–2016 EVD outbreak in Sierra Leone and explored the association of antibody seropositivity and concentration with potential risk factors for EBOV infection.

## The Study

We conducted a seroprevalence study in Kambia District, Sierra Leone, during March 2016–June 2018. We nested the study within the screening visit of the EBOVAC-Salone (https://www.ebovac.org) randomized controlled trial (RCT), which evaluated the safety and immunogenicity of the 2-dose Ad26.ZEBOV, MVA-BN-Filo Ebola vaccine regimen (ClinicalTrials.gov, no. NCT02509494) (*2*,*3*). Persons who reported having a previous EVD diagnosis and persons who previously received a candidate Ebola vaccine were ineligible for the RCT, and we excluded them from the seroprevalence study. We recruited adults first, then recruited children in 3 age cohorts: 12–17, 4‒11, and 1‒3 years of age.

We measured IgG to EBOV glycoprotein (GP) by using the Filovirus Animal Non-Clinical Group (FANG) ELISA (Q2 Solutions Vaccine Testing Laboratory, https://www.q2labsolutions.com). We determined seropositivity by using a cutoff of >607 ELISA units (EU)/mL, which was calculated previously in an EBOV-naive population in West Africa (*4*) (Appendix). 

Among 1,282 study participants (Figure), 687 (53.6%) were <18 years of age (median 16 years, IQR 7–25 years), and 827 (64.5%) were male. Among 1,272 participants with antibody results, we considered 107 (8.4%, 95% CI 7.0%–10.0%) seropositive for EBOV GP IgG by using the prespecified cutoff.

**Figure:**
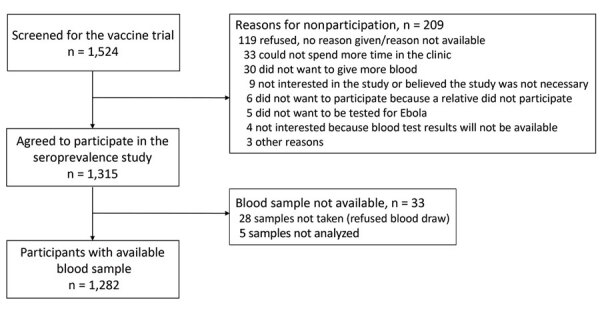
Flow chart of participants screened for the Ebola virus vaccine trial and subsequent seropositivity study of community members affected by the 2014–2016 Ebola outbreak, Sierra Leone.

Risk factor analysis showed that, after adjusting for age and sex, the only characteristic associated with seropositivity was living in a household compound with >1 pigs during the outbreak (adjusted odds ratio [OR] 4.5, 95% CI 1.6–13.0; p = 0.01) (Tables 1, Tables 2; Appendix Table 1). The EBOV antibody geometric mean concetration (GMC) was higher in participants ≥5 years of age than in younger children (Appendix Table 1). After adjusting for age and sex, only pig ownership remained associated with antibody concentration (adjusted GMC ratio 3.0, 95% CI 1.5–5.9; p<0.01) (Table 2).

**Table 1:** Potential EVD exposure in community or work during the 2014–2016 EVD outbreak and antibody seropositivity and GMC among participants in a study of EBOV GP–specific binding antibody seropositivity, Sierra Leone*

Risk factors	No. (%), n = 1,282	No. seropositive/ no. tested (%)	OR (95% CI)	Adjusted OR (95% CI)†	GMC, EU/mL (95% CI)	GMC ratio (95% CI)	Adjusted GMC ratio (95% CI)†
Living in a village or town with Ebola cases, n = 1,281							
N	199 (15.5)	10/198 (5.1)	Referent, p = 0.049	Referent, p = 0.125	49 (40–58)	Referent, p = 0.010	Referent, p = 0.882
Y	1,082 (84.5)	97/1,073 (9.0)	1.9 (1.0–3.6)	1.7 (0.8–3.3)	65 (60–71)	1.3 (1.1–1.6)	1.0 (0.8–1.3)
Knowing someone who had Ebola							
No, don't know	1,044 (81.4)	82/1,036 (7.9)	Referent, p = 0.193		61, 56–67)	Referent, p = 0.204	
Y	238 (18.6)	25/236 (10.6)	1.4 (0.9–2.2)		70 (57–85)	1.1 (0.92–1.4)	
No. EVD cases known by participant							
0	1,044 (81.4)	82/1,036 (7.9)	Referent, p = 0.55		61 (56–67)	Referent, p = 0.382	
1	125 (9.8)	13/125 (10.4)	1.4 (0.7–2.5)		64 (49–85)	1.1 (0.8–1.4)	
2–3	66 (5.2)	8/65 (12.3)	1.6 (0.8–3.5)		84 (57–124)	1.4 (0.9–2.0)	
>3	47 (3.7)	4/46 (8.7)	1.1 (0.4–3.2)		66 (44–99)	1.1 (0.7–1.6)	
Closest relationship with an EVD case, n = 1,280							
No relationship‡	1,044 (81.5)	82/1,036 (7.9)	Referent, p = 0.197		61, 56–67)	Referent, p = 0.259	
Close family§	27 (2.1)	1/27 (3.7)	0.5 (0.1–3.3)		52 (33–81)	0.9 (0.5–1.3)	
Other relative	52 (4.1)	6/51 (11.8)	1.6 (0.6–3.7)		64 (42–96)	1.0 (0.7–1.6)	
Friend	59 (4.6)	4/59 (6.8)	0.8 (0.3–2.4)		64 (45–91)	1.1 (0.7–1.5)	
Community member	98 (7.7)	14/97 (14.4)	2.0 (1.1–3.7)		86 (62–120)	1.4 (1.0–2.0)	
Living in the same household with an EVD case, n = 1,280							
N	1,269 (99.1)	107/1,260 (8.5)	–		63 (58–68)	Referent, p = 0.814	
Y	11 (0.9)	0/10 (0.0)	–		56 (31–102)	0.9 (0.5–1.6)	
Caring for an EVD case, n = 1,281							
N	1,272 (99.3)	107/1,262 (8.5)	–		63 (58–68)	Referent, p = 0.600	
Y	9 (0.7)	0/9 (0.0)	–		48 (24–98)	0.8 (0.4–1.6)	
Direct body contact with an EVD case, n = 1,281							
N	1,275 (99.5)	107/1,265 (8.5)	–		62 (57–67)	Referent, p = 0.640	
Y	6 (0.5)	0/6 (0.0)	–		83 (28–242)	1.3 (0.5–3.9)	
Attending a funeral of an EVD case							
N	1,263 (98.5)	105/1,254 (8.4)	Referent, p = 0.691		62 (57–67)	Referent, p = 0.346	
Y	19 (1.5)	2/18 (11.1)	1.4 (0.3–6.0)		87 (37–204)	1.4 (0.6–3.3)	
Healthcare frontline worker during EVD outbreak							
No, NA¶	1,254 (97.8)	105/1,244 (8.4)	Referent, p = 0.802		63 (58–69)	Referent, p = 0.798	
Y	28 (2.2)	2/28 (7.1)	0.8 (0.2–3.6)		58 (36–93)	0.9 (0.6–1.5)	

*Seropositivity defined as >607 EU/mL. EBOV GP–specific binding antibodies were indeterminate in 10 participants. p values calculated by using likelihood ratio test. EBOV GP, Ebola virus glycoprotein; EU, ELISA units; EVD, Ebola virus disease; GMC, geometric mean concentration; NA, not applicable; OR, odds ratio. †Adjusted for age and sex. ‡Participant did not know anyone with Ebola. §Participant was the parent or child or spouse or sibling of an EVD case. ¶Not applicable because participant was a child or did not have a job.

**Table 2:** Potential risk factors for transmission of Ebola virus from animals during the 2014–2016 EVD outbreak and antibody seropositivity and GMC among participants in a study of EBOV GP–specific binding antibody seropositivity, Sierra Leone*

Risk factors	No. (%), n = 1,282	No. seropositive/ no. tested (%)	OR (95% CI)	Adjusted OR (95% CI)†	GMC, EU/mL (95% CI)	GMC ratio (95% CI)	Adjusted GMC ratio (95% CI)†
Number of domestic animals in the participant’s compound							
0	503 (39.2)	45/498 (9.0)	Referent, p = 0.558		59 (51–67)	Referent, p = 0.462	
1–5	374 (29.2)	33/371 (8.9)	1.0 (0.6–1.6)		65 (55–75)	1.1 (0.9–1.3)	
>5	405 (31.6)	29/403 (7.2)	0.8 (0.5–1.3)		66 (57–76)	1.1 (0.9–1.3)	
Having the following domestic animals in the compound‡							
Dog							
N	1,116 (87.1)	90/1,107 (8.1)	Referent, p = 0.349		66 (52–84)	Referent, p = 0.559	
Y	165 (12.9)	17/164 (10.4)	1.3 (0.8–2.3)		62 (57–67)	1.1 (0.8–1.4)	
Cat							
N	951 (74.2)	80/943 (8.5)	Referent, p = 0.887		61 (56–67)	Referent, p = 0.400	
Y	330 (25.8)	27/328 (8.2)	1.0 (0.6–1.5)		66 (56–78)	1.1 (0.9–1.3)	
Goat, sheep							
N	870 (67.9)	76/863 (8.8)	Referent, p = 0.465		62 (56–68)	Referent, p = 0.781	
Y	411 (32.1)	31/408 (7.6)	0.9 (0.6–1.3)		62 (57–67)	1.0 (0.9–1.2)	
Pig							
N	1,263 (98.6)	102/1,253 (8.1)	Referent, p = 0.015	Referent, p = 0.014	61 (57–67)	Referent, p<0.001	Referent, p = 0.001
Y	18 (1.4)	5/18 (27.8)	4.3 (1.5–12.4)	4.5 (1.6–13.0)	200 (93–431)	3.3 (1.5–7.1)	3.0 (1.5–5.9)
Other							
N	825 (64.4)	73/817 (8.9)	Referent, p = 0.370		61 (55–68)	Referent, p = 0.513	
Y	456 (35.6)	34/454 (7.5)	0.8 (0.5–1.3)		65 (57–74)	1.1 (0.9–1.3)	
Touching sick or dead domestic animals							
N	1,253 (97.7)	106/1,243(8.5)	Referent, p = 0.275		63 (58–68)	Referent, p = 0.824	
Y	29 (2.3)	1/29 (3.5)	0.4 (0.1–2.8)		59 (36–97)	0.9 (0.6–1.6)	
Hunting for wild animals§							
N	1,261 (99.3)	105/1,251(8.4)	Referent, p = 0.779		63 (58–68)	Referent, p = 0.859	
Y	9 (0.7)	1/9 (11.1)	1.4 (0.2–11.0)		57 (17–191)	0.9 (0.3–3.1)	
Touching sick or dead wild animals							
N	1,277 (99.6)	106/1,267 (8.4)	Referent, p = 0.419		62 (58–68)	Referent, p = 0.825	
Y	5 (0.4)	1/5 (20.0)	2.7 (0.3–24.7)		54 (8–369)	0.9 (0.1–5.9)	
Consuming bushmeat							
N	1,275 (99.4)	106/1,265 (8.4)	Referent, p = 0.606		62 (58–68)	Referent, p = 0.962	
Y	7 (0.6)	1/7(14.3)	1.8 (0.2–15.3)		61 (14–274)	1.0 (0.2–4.4)	

*Seropositivity defined as >607 EU/mL. EBOV GP–specific binding antibodies were indeterminate in 10 participants. p values calculated by using likelihood ratio test. EBOV, Ebola virus; EU, ELISA units; GMC, geometric mean concentration; GP, glycoprotein; OR, odds ratio. †Adjusted for age and sex. ‡Participants could indicate >1 type of domestic animal. §Types of wild animals hunted by participants who answered yes included monkeys, duiker antelopes, bats, and rodents.

The 8.4% seroprevalence in our study is within the range of estimates (0%–24%) from prior studies; however, this range is large because of the use of different assays, different seroprevalence thresholds, different levels of exposure to EVD cases, and studies undertaken in different geographic areas and at different timepoints relative to reported outbreaks (*1*). Our estimate is similar to the baseline EBOV antibody seroprevalence (4.0%) measured in another Ebola vaccine trial conducted in Liberia during the 2014–2016 EVD outbreak that used the same assay and cutoff (*5*).

Similar to results from previous studies, our findings showed a statistically significant increase in EBOV antibody concentration with participants’ age in our study, possibly because of increased exposure of older age groups to EBOV or to other infections that could induce cross-reactive antibodies to the EBOV GP (*6*,*7*). Potential exposures to EVD, such as healthcare work, contact with EVD cases, and funeral attendance, which were associated with EBOV transmission in other studies (*8*), were not associated with EBOV antibody seropositivity or concentration in our study. However, few participants reporting those risk factors, and our study might have lacked the power to detect such associations.

We found an independent association of both EBOV antibody seropositivity and concentration with residence in a household compound that owned >1 pigs during the Ebola outbreak. Pigs can be experimentally infected with EBOV and can transmit the virus to nonhuman primates (*9*). EBOV-specific antibodies have been found in pigs in Sierra Leone and Guinea, suggesting that pigs can be naturally infected by EBOV (*10*,*11*). Pigs in the Philippines have been found to be naturally infected with Reston virus, an EBOV strain that is not known to cause disease in humans. Reston virus–specific antibodies were found in healthy farmers in contact with the infected pigs, suggesting potential transmission from pigs to humans (*12*). However, we found no association of EBOV antibody with having other domestic animals, in particular dogs, which also could be infected with EBOV (*13*,*14*).

One strength of our study is that we conducted our study in an area with prolonged EBOV transmission during the 2014–2016 EVD outbreak. Further, we explored a wide range of potential risk factors for EBOV acquisition, and we used the FANG ELISA, which has been proven to be more precise and accurate than a commercial alternative (*4*).

The first limitation of our study is that the parent RCT did not require random sampling of potential participants’ households, which could have affected the generalizability of our results to the general population. The RCT recruitment was age-staggered, and the youngest age cohort (1‒3 years of age) was recruited >2 years after the EVD outbreak ended. However, a sensitivity analysis suggested that year of recruitment had a negligible confounding effect on the lower EBOV antibody concentrations observed in the youngest children (Appendix Table 2). Our study was conducted at the end of the 2014–2016 EVD outbreak in Sierra Leone, when public health measures to contain EBOV transmission had been in place for several months and the population had received messages about EVD prevention. This factor could have caused an underreporting of behaviors considered to put persons at risk for EVD. For example, hunting and consumption of bushmeat was rarely reported by our participants, in contrast with some reports that describe frequent hunting and bushmeat consumption in West Africa (*15*). The association of both antibody seropositivity and concentration with pig ownership is based on only 18 participants who reported keeping >1 pigs in their household compound at the time of the outbreak. This association could have occurred by chance, although the evidence of an association is quite strong. The observed association also could be confounded by unrecorded risk factors among participants who also kept pigs, such as EBOV transmission clustering in participants from a household that also owned pigs. However, that possibility seems unlikely because none of the seropositive participants who owned pigs reported contact with an EVD case, and these participants all came from different households. Finally, we are not able to determine whether EBOV antibody seropositivity in this setting reflects true asymptomatic infection because we cannot exclude underreporting of earlier EVD symptoms and we have not yet investigated cross-reactivity with other viral infections. Whether EBOV seropositivity reflects acquired immunity that might provide some protection against future EBOV infections also is unclear.

Our findings suggest that the role of pigs as potential, occasional reservoirs of EBOV needs to be investigated further. The presence of antibodies binding the EBOV GP could also suggest circulation of other infectious agents, probably viruses, inducing cross-reactivity with the EBOV GP, but this possibility needs further investigation.

## Conclusions

The incidence of EBOV infection during the 2014–2016 EVD outbreak in Sierra Leone could have been higher than previously reported; 8.4% of adults and children from a community affected by the outbreak who never experienced symptoms of EVD had serologic responses to EBOV above a cutoff threshold. Our study suggests that EBOV might cause asymptomatic infection, but whether underreporting of symptoms, FANG assay specificity, or exposure to other viral infections that could generate cross-reactive antibodies also contributed to the results is unclear. These questions would benefit from further investigation to help define the extent of future EVD outbreaks. Countries at high risk for EVD outbreaks should be aware of the risk of asymptomatic or paucisyntomatic infections. 

Appendix Additional information on Ebola virus seroprevalence study in a community previously affected by Ebola, Sierra Leone.
