# Supplementary material for: Ebola Virus Glycoprotein IgG Seroprevalence in Community Previously Affected by Ebola, Sierra Leone
Source: Emerg Infect Dis. 2022 Mar;28(3):734–8. doi: 10.3201/eid2803.211496 (PMC8888237; doi:10.3201/eid2803.211496)
Supplement: Appendix — Additional information on Ebola virus seroprevalence study in a community previously affected by Ebola, Sierra Leone. [file 21-1496-Techapp-s1.pdf]

Article DOI: https://doi.org/10.3201/eid2803.211496

Ebola Virus Glycoprotein IgG Seroprevalence in Community Previously Affected by Ebola, Sierra Leone

Appendix

Methods

Study Design

We conducted a cross-sectional seroprevalence study of immunoglobulin G (IgG) antibodies against Ebola virus (EBOV) glycoprotein (GP) during March 16, 2016–June 29, 2018. We nested the study within the screening visit of the EBOVAC-Salone (https://www.ebovac.org) randomized controlled trial (RCT), which was being conducted to evaluate the safety and immunogenicity of a 2-dose heterologous vaccination regimen with Ad26.ZEBOV and MVA-BN-Filo Ebola vaccines (protocol no. VAC52150EBL3001; ClinicalTrials.gov no. NCT02509494).

Study Participants

We enrolled participants from 3 sites in Kambia District, northern Sierra Leone; 2 sites in Kambia town and 1 site in the neighboring community of Rokupr, a rural village ≈15 km from Kambia town. Both areas were affected by widespread and prolonged EBOV transmission during the Ebola virus disease (EVD) epidemic in West Africa (*1*).

We recruited adults first, during March 16, 2016–December 29, 2016; then we enrolled children during March 21 2017–June 29, 2018 in 3 age cohorts: 12–17, 4–11, and 1–3 years of age. We counselled potential participants on the importance of providing accurate medical information, including any history of EVD, close contact with a person who had EVD, or prior vaccination with a candidate Ebola vaccine. Persons who reported having an EVD diagnosis in the past or who previously had been vaccinated with a candidate Ebola vaccine were considered ineligible for both the RCT and we did not include them in the seroprevalence study.

We obtained informed consent from adult participants and from parents or guardians for participants who were <18 years of age. We also asked children $\geq$7 years of age to give their assent for participation. Ethical approval for the study was received from the Sierra Leone Ethics Committee and the London School of Hygiene & Tropical Medicine (LSHTM) Ethics Committee (reference no. 10537).

Study Procedures

We interviewed study participants to collect information on potential risk factors for EBOV infection, including residence in areas where EVD cases occurred during the 2014–2016 outbreak, healthcare work during the outbreak, travel, contact with EVD cases, funeral attendance, and contact with or consumption of wild animals. Because EBOV also is known to infect domestic animals, including dogs and pigs, we also collected information on contacts with these animals during the outbreak (*2–7*).

Approximately 2 mL of blood was collected at enrollment. Samples were left to clot for 30 minutes, then centrifuged at 1,500 g (rpm) for 10 min at the study clinics. At the research laboratory, we aliquoted serum and froze it at −20°C. We stored serum samples at −20°C until shipped in controlled temperature containers to the laboratory in the United States for sample analysis. Q2 Solutions Vaccine Testing Laboratory (https://www.q2labsolutions.com) measured IgG against EBOV GP by using the EBOV GP Filovirus Animal Non-Clinical Group (FANG) ELISA. Validation of the FANG ELISA was endorsed by the US Food and Drug Administration (FDA) in February 2017 (Q2 Solutions, pers. comm., 2017. FANG ELISA has a lower limit of quantification (LLOQ), 36.11 ELISA units (EU)/mL, and has no established cutoff to distinguish seropositive persons after EBOV infection from seronegative persons (Q2 Solutions, pers. comm., 2019). To determine seropositivity, we used a cutoff of >607 EU/mL, which was calculated in a previous study using serum samples collected from 100 EBOV-naive persons from Mali during 2004–2011 and was defined as the antibody titer of 3 SD above the mean ($\log_{10}$ transformed) (*8*). This cutoff was considered appropriate to provide an estimate of the prevalence of IgG to EBOV GP in a setting in West Africa. We also conducted a post-hoc analysis with an alternative cutoff calculated by using serum samples from 388 EBOV-naive persons from the United Kingdom (See Alternative cutoff calculation).

Sample Size and Statistical Analysis

We did not conduct a formal sample size calculation for this study because the number of enrolled participants was determined by the number of participants screened for the RCT. We also had limited data on the estimated prevalence of IgG to EBOV GP in the general population afer an EVD outbreak. However, we estimated that a sample of 1,250 persons would enable us to estimate a prevalence of 1.0% with a precision of approximately ±0.55% (i.e., 95% CI 0.45%–1.55%).

We conducted our statistical analysis for all participants with an available FANG ELISA result. We calculated the seroprevalence of IgG to EBOV GP as a percentage of study participants who had an antibody concentration above the prespecified cutoff of >607 EU/mL. We obtained the antibody geometric mean concentration (GMC) and 95% CI by calculating the mean and 95% CI of the log-transformed values, and then transforming these results back into the original units by taking the antilogs. To calculate GMC, we imputed values below the LLOQ as LLOQ/2 (18.055 EU/mL). We calculated the odds ratio (OR) and 95% CI to measure the association between potential risk factors for acquisition of EBOV and seropositivity, using logistic regression. We calculated the GMC ratio and 95% CI to measure the association between the same risk factors and IgG antibody concentration, using linear regression. For the risk factor analysis, we selected a total of 26 variables out of 47 questions related to risk factors or potential confounders obtained from participants' interviews. Among those questions, we used 11 questions about household characteristics (ownership of goods, such as television, radio, etc.) to calculate the socioeconomic status of the household with principal component analysis. We adjusted the multivariable analyses for age and sex (a priori confounding factors). We conducted a post-hoc sensitivity analysis adjusting for year of enrollment to explore whether the age distribution of the EBOV GP antibody concentration could have been influenced by the age-staggered recruitment procedure. We used Stata 16 (StataCorp LLC, https://www.stata.com) for all the statistical analyses.

Alternative Cutoff Calculation

In a post-hoc analysis, we calculated an alternative seropositivity cutoff by using baseline Ebolavirus IgG levels from 388 healthy persons from the United Kingdom who were enrolled in an Ebola vaccine trial (protocol no. VAC52150EBL2001) during 2014–2015 (*9*). The investigators of this study conducted the sample analysis by using the FANG ELISA at Q2

Solutions Vaccine Testing Laboratory. Among the 388 participants, 26 had a baseline result above the LLOQ of 36.11 EU/mL. We imputed values below the LLOQ as LLOQ/2 (18.055 EU/mL). We defined the seropositivity cutoff as the antilog value of 3 SD above the mean of the $\log_{10}$ transformed values, as calculated in a previous study (*8*).

The EBOV GP antibody GMC in the 388 EBOV-naive persons from the UK was 20.44 EU/mL, with a geometric standard deviation (GSD) of 1.69 EU/mL. To calculate seropositivity cutoff we use the formula: $GMC \times (GSD)^3$.

Seropositivity cutoff = $20.44 \times (1.69)^3 = 99.03$ EU/mL

Results

Detailed Description of Study Results

A total of 1,524 potential participants were screened for the VAC52150EBL3001 trial, of whom 1,315 (86.3%) agreed to participate in the seroprevalence study (Figure). Blood samples were available for 1,282 (97.5%) participants, 687 (53.6%) of whom were aged <18 years (median age 16 years, IQR = 7–25 years) and 827 (64.5%) of whom were male (Appendix Table 1).

Only 238 (18.6%) participants reported that they knew someone who had EVD during the outbreak (Table 1). Eleven (0.9%) participants reported that someone in their household had experienced EVD and 9 (0.7%) participants cared for someone with EVD. Six (0.5%) participants had direct body contact with an EVD patient. Only 28 participants (2.2%) undertook healthcare or frontline (i.e., burial team) work during the EVD outbreak. Only 9 (0.7%) reported hunting for wild animals and only 7 (0.6%) said that they had consumed bushmeat (Table 2).

Because the FANG ELISA results were indeterminate in 10 of the 1,282 samples, the estimation of IgG seroprevalence and GMC were based on results from 1,272 participants. Of those 1,272 samples, 684 (53.8%) had a result that was above the LLOQ of 36.11 EU/mL for the FANG ELISA. Overall, 107 participants (8.4%, 95% CI 7.0%–10.0%) had a result above the prespecified seropositivity cutoff of 607 EU/mL and we considered these samples to be seropositive for EBOV GP in our study.

There were fewer seropositive participants among children <5 years compared with older age groups (Appendix Table 1). However, we found no statistical evidence of an association between seropositivity and age. We also saw no statistically significant difference in the percentage of seropositive samples by sex. In univariable analyses, we noted some evidence of an association between seropositivity and living in a village or town with EVD cases (Table 1), or in a household compound with ≥1 pigs at the time of the outbreak (Table 2). After adjusting for age and sex, only having ≥1 pigs in the household compound at the time of the outbreak remained associated with EBOV seropositivity (adjusted OR 4.5, 95% CI 1.6–13.0, $p = 0.01$) (Table 2). A post-hoc analysis with an alternative cutoff calculated by using serum samples from 388 EBOV-naive persons from the United Kingdom, showed similar results (see Alternative Cutoff Analysis results).

We noted a statistically significant increase in EBOV GP binding antibody GMC with age and GMC was higher in participants ≥5 years of age than in younger children (Appendix Table 1). This association remained after adjusting for year of recruitment, which suggested that it was not due to the age-staggered recruitment process (Appendix Table 2). Male persons had a slightly higher GMC than female persons but we saw no evidence of a difference after adjusting for age. Other statistically significant variables associated with EBOV GP binding antibody concentration on univariable analysis were education, frequency of travel outside the place of residence, living in a village or town with EVD cases, and having ≥1 pigs in the household compound at the time of the outbreak (Table 1, Table 2; Appendix Table 1). After adjusting for age and sex, we saw no evidence of an association between antibody concentration and education or travel or residence in a village or town with EVD cases. However, we still saw evidence of an association between antibody concentration and the presence of ≥1 pigs in the household compound at the time of the outbreak (adjusted GMC ratio 3.0, 95% CI 1.5%–5.9%, $p < 0.01$) (Table 2).

Alternative Cutoff Analysis Results

Because the assay has no established diagnostic serostatus threshold, we calculated a range of seropositivity estimates by using different cut-off values and the prespecified cutoff used in our study (Appendix Table 3). We also conducted a post-hoc analysis with an alternative cutoff calculated by using serum samples from EBOV-naive persons from the United Kingdom (see Alternative Cutoff Calculation). Overall, 411 participants (32.3%, 95% CI 29.7%–34.9%)

had a result above the seropositivity cutoff of 99.03 EU/mL and we considered these samples to be seropositive for EBOV GP in our supplementary analysis.

The number of seropositive participants increased with age and fewer children <5 years of age were seropostive compared with persons in older age groups (Appendix Table 4). We saw no statistically significant difference in the percentage of seropositive participants by sex. In univariable analyses, we noted some evidence of an association between seropositivity and education and living in a household compound that kept ≥1 pigs at the time of the outbreak (Appendix Tables 4–6). After adjusting for age and sex, only having ≥1 pigs in the household compound at the time of the outbreak remained associated with EBOV seropositivity (adjusted OR 4.1, 95% CI 1.5–11.4, $p < 0.01$) (Appendix Table 6).

Discussion

FANG ELISA Uses and Limitations

The FANG ELISA used in our study has been proven to be more precise and accurate than a commercial alternative for the assessment of immune response after Ebola vaccination (*8*). Despite being the best option available at the time, the assay has some limitations. Positivity has been observed in samples from countries that have never experienced EBOV outbreaks, which indicates that the assay might not have a high specificity (*10–13*). For this reason, we adopted a seropositivity cutoff that has been calculated in EBOV-naive persons from West Africa, although this analysis was not done in the same laboratory where our study samples were analyzed (*8*). Another limitation of the FANG ELISA is that it only detects IgG against the EBOV GP, but a concomitant test to detect IgG against the EBOV nucleoprotein could have enabled a better identification of previous EBOV infections, as noted in another study (*14*). A seropositive cutoff of >607 EU/mL could be considered high for a seroepidemiologic study, considering that in some Ebola vaccine trials the antibody concentration that was achieved post vaccination was sometimes below this threshold, even in participants considered as vaccine responders (*10–13*). However, we believe that this cutoff is suitable to provide a conservative estimate of the prevalence of IgG to EBOV GP in West Africa but it would not be appropriate to use this cutoff for the interpretation of post-vaccination results in a clinical trial. Most Ebola vaccine trials that used the FANG ELISA for the measurement of postvaccination antibody response have adopted

a vaccine responder definition that was based on an x-fold increase over prevaccination baseline values, instead of using a predefined cutoff (*10–13*). We are aware that, without an established diagnostic serostatus threshold, the choice of a cutoff can be arbitrary. Thus, we also analyzed the data as a continuous variable, i.e., EBOV IgG concentration and we conducted a post-hoc analysis using an alternative cutoff calculated in EBOV-naive persons from the United Kingdom and these analyses showed similar results.

References

1. World Health Organiztion. Ebola maps from 30 March 2016: Geographical distribution of new and total confirmed cases [cited 2021 Mar 1]. http://apps.who.int/ebola/sites/default/files/thumbnails/image/sitrep_casecount_40.png?ua=1
2. Allela L, Bourry O, Pouillot R, Délicat A, Yaba P, Kumulungui B, et al. Ebola virus antibody prevalence in dogs and human risk. Emerg Infect Dis. 2005;11:385–90.
3. Haun BK, Kamara V, Dweh AS, Garalde-Machida K, Forkay SSE, Takaaze M, et al. Serological evidence of Ebola virus exposure in dogs from affected communities in Liberia: A preliminary report. PLoS Negl Trop Dis. 2019;13(7):e0007614.
4. Gumusova S, Sunbul M, Leblebicioglu H. Ebola virus disease and the veterinary perspective. Ann Clin Microbiol Antimicrob. 2015;14:30.
5. Fischer K, Camara A, Troupin C, Fehling SK, Strecker T, Groschup MH, et al. Serological evidence of exposure to ebolaviruses in domestic pigs from Guinea. Transbound Emerg Dis. 2020;67:724–32.
6. Fischer K, Jabaty J, Suluku R, Strecker T, Groseth A, Fehling SK, et al. Serological evidence for the circulation of ebolaviruses in pigs from Sierra Leone. J Infect Dis. 2018;218:S305–11.
7. Weingartl HM, Embury-Hyatt C, Nfon C, Leung A, Smith G, Kobinger G. Transmission of Ebola virus from pigs to non-human primates. Sci Rep. 2012;2:811.
8. Logue J, Tuznik K, Follmann D, Grandits G, Marchand J, Reilly C, et al. Use of the Filovirus Animal Non-Clinical Group (FANG) Ebola virus immuno-assay requires fewer study participants to power a study than the Alpha Diagnostic International assay. J Virol Methods. 2018;255:84–90.
9. Pollard AJ, Launay O, Lelievre JD, Lacabaratz C, Grande S, Goldstein N, et al.; EBOVAC2 EBL2001 Study Group. Safety and immunogenicity of a two-dose heterologous Ad26.ZEBOV and MVA-BN-Filo Ebola vaccine regimen in adults in Europe (EBOVAC2): a randomised, observer-blind, participant-blind, placebo-controlled, phase 2 trial. Lancet Infect Dis. 2021;21:493–506.

10. Mutua G, Anzala O, Luhn K, Robinson C, Bockstal V, Anumendem D, et al. Safety and immunogenicity of a 2-dose heterologous vaccination regimen with Ad26.ZEBOV and MVA-BN-Filo Ebola vaccines: 12-month data from a phase 1 randomized clinical trial in Nairobi, Kenya. J Infect Dis. 2019;220:57–67.

11. Anywaine Z, Whitworth H, Kaleebu P, Praygod G, Shukarev G, Manno D, et al. Safety and immunogenicity of a 2-dose heterologous vaccination regimen with Ad26.ZEBOV and MVA-BN-Filo Ebola vaccines: 12-month data from a phase 1 randomized clinical trial in Uganda and Tanzania. J Infect Dis. 2019;220:46–56.

12. Afolabi MO, Ishola D, Manno D, Keshinro B, Bockstal V, Rogers B, et al. Safety and immunogenicity of the two-dose heterologous Ad26.ZEBOV and MVA-BN-Filo Ebola vaccine regimen in children in Sierra Leone: a randomised, double-blind, controlled trial. Lancet Infect Dis. 2022;22:110–22.

13. Ishola D, Manno D, Afolabi MO, Keshinro B, Bockstal V, Rogers B, et al. Safety and long-term immunogenicity of the two-dose heterologous Ad26.ZEBOV and MVA-BN-Filo Ebola vaccine regimen in adults in Sierra Leone: a combined open-label, non-randomised stage 1, and a randomised, double-blind, controlled stage 2 trial. Lancet Infect Dis. 2022;22:97–109.

14. Richardson E, Kelly J, Barrie M, Mesman A, Karku S, Quiwa K, et al. Minimally symptomatic infection in an Ebola 'hotspot': a cross-sectional serosurvey. PLoS Negl Trop Dis. 2016;10(11):e0005087.

Appendix Table 1. Sociodemographic characteristics, Ebola virus glicoprotein-specific binding antibody seropositivity, and geometric mean concentration among participants in a study of EBOV GP–specific binding antibody seropositivity, Sierra Leone*

Characteristics	No. (%), n = 1,282	No. seropositive/no. tested (%)	OR (95% CI)	Adjusted OR (95% CI)†	GMC, EU/mL (95% CI)	GMC ratio (95% CI)	Adjusted GMC ratio (95% CI)†
Age group, y							
1–4	243 (19.0)	14/240 (5.8)	Referent, 1.0 (p = 0.184)	Referent, 1.0 (p = 0.165)‡	32 (26–38)	Referent, 1.0 (p<0.001)	Referent, 1.0 (p<0.001)‡
5–9	170 (13.3)	18/168 (10.7)	1.9 (0.9–4.0)	1.9 (0.9–4.0)	69 (54–88)	2.2 (1.6–2.9)	2.2 (1.6–2.9)
10–19	354 (27.6)	24/353 (6.8)	1.2 (0.6–2.3)	1.2 (0.6–2.3)	71 (61–82)	2.2 (1.8–2.8)	2.2 (1.8–2.8)
20–39	390 (30.4)	39/387 (10.1)	1.8 (1.0–3.4)	1.9 (1.0–3.6)	77 (66–90)	2.4 (1.9–3.0)	2.3 (1.9–2.9)
≥40	125 (9.7)	12/124 (9.7)	1.7 (0.8–3.9)	1.8 (0.8–3.9)	72 (56–92)	2.3 (1.7–3.1)	2.2 (1.7–3.0)
Sex							
F	455 (35.5)	39/451 (8.7)	Referent, 1.0 (p = 0.823)	Referent, 1.0 (p = 0.560)‡	54 (48–62)	Referent, 1.0 (p = 0.018)	Referent, 1.0 (p = 0.125)‡
M	827 (64.5)	68/821 (8.3)	1.0 (0.6–1.4)	0.9 (0.6–1.3)	67 (61–74)	1.2 (1.0–1.5)	1.1 (1.0–1.4)
Highest education level							
No education	362 (28.2)	25/360 (6.9)	Referent, 1.0 (p = 0.572)		42 (37–49)	Referent, 1.0 (p<0.001)	Referent, 1.0 (p = 0.653)
Primary, grades 1–6	378 (29.5)	35/374 (9.4)	1.4 (0.8–2.4)		74 (63–86)	1.7 (1.4–2.2)	1.1 (0.8–1.5)
Secondary school	480 (37.4)	43/477 (9.0)	1.3 (0.8–2.2)		72 (63–82)	1.7 (1.4–2.1)	1.0 (0.7–1.3)
College, university	62 (4.9)	4/61 (6.6)	0.9 (0.3–2.8)		73 (54–100)	1.7 (1.2–2.4)	0.9 (0.6–1.5)
Household socioeconomic status							
Low	470 (36.7)	36/464 (7.8)	Referent, 1.0 (p = 0.805)		57 (50–66)	Referent, 1.0 (p = 0.294)	
Middle	396 (30.9)	34/394 (8.6)	1.1 (0.7–1.8)		66 (57–76)	1.1 (0.9–1.4)	
High	416 (32.5)	37/414 (8.9)	1.2 (0.7–1.9)		66 (57–75)	1.1 (0.9–1.4)	
Number of persons (adults and children) in the household, n = 1,276							
<5	274 (21.5)	21/270 (7.8)	Referent, 1.0 (p = 0.769)		59 (49–70)	Referent, 1.0 (p = 0.300)	
5–9	529 (41.4)	48/527 (9.1)	1.2 (0.7–2.0)		68 (59–77)	1.2 (0.9–1.4)	
≥10	473 (37.1)	38/469 (8.1)	1.0 (0.6–1.8)		60 (53–68)	1.0 (0.8–1.3)	
Number of children in the household, n = 1,274							
0–2	466 (36.6)	41/463 (8.9)	Referent, 1.0 (p = 0.646)		64 (56–73)	Referent, 1.0 (p = 0.854)	
3–5	536 (42.1)	40/530 (7.6)	0.8 (0.5–1.3)		61 (54–69)	1.0 (0.8–1.1)	
>5	272 (21.3)	25/271 (9.2)	1.0 (0.6–1.8)		64 (54–75)	1.0 (0.8–1.2)	
Frequency of travel outside of village or town of residence, n = 1,276							
Never traveled	510 (40.0)	33/507 (6.5)	Referent, 1.0 (p = 0.186)		55 (49–63)	Referent, 1.0 (p = 0.042)	Referent, 1.0 (p = 0.578)
Every day	19 (1.5)	2/19 (10.5)	1.7 (0.4–7.6)		110 (59–204)	2.0 (1.0–3.7)	1.5 (0.8–3.0)
≥1×/wk	58 (4.5)	9/58 (15.5)	2.6 (1.2–5.8)		90 (57–142)	1.6 (1.0–2.6)	1.3 (0.8–1.9)
≥1×/mo.	235 (18.4)	21/232 (9.1)	1.4 (0.8–2.5)		68 (56–82)	1.2 (1.0–1.5)	1.0 (0.8–1.3)
<1×/mo.	454 (35.6)	41/450 (9.1)	1.4 (0.9–2.3)		63 (55–72)	1.1 (0.9–1.4)	1.0 (0.8–1.3)

*Seropositivity defined as >607 EU/mL. p values calculated by using likelihood ratio test. EBOV–GP, Ebola virus glycoprotein; EVD, Ebola virus disease; GMC, geometric mean concentration; OR, odds ratio. EBOV, Ebola virus; EU, ELISA units;.
†Adjusted for age and sex.
‡Age adjusted for sex. Sex adjusted for age.

Appendix Table 2. Association between antibody concentration and age at recruitment, before and after adjusting for year of recruitment, among participants in a study of EBOV GP–specific binding antibody seropositivity, Sierra Leone*

Characteristics	No. (%), n = 1,282	GMC, EU/mL (95% CI)	GMC ratio (95% CI)	Adjusted GMC ratio (95% CI)
Age group, y				
1–4	243 (19.0)	32 (26–38)	Referent, 1.0 (p<0.001)	Referent, 1.0 (p<0.001)†
5–9	170 (13.3)	69 (54–88)	2.2 (1.6–2.9)	2.1 (1.5–2.9)
10–19	354 (27.6)	71 (61–82)	2.2 (1.8–2.8)	2.1 (1.5–2.9)
20–39	390 (30.4)	77 (66–90)	2.4 (1.9–3.0)	2.2 (1.4–3.5)
≥40	125 (9.7)	72 (56–92)	2.3 (1.7–3.1)	2.1 (1.3–3.4)
Year of recruitment				
2016	595 (46.4)	75 (67–85)	Referent, 1.0 (p<0.001)	Referent, 1.0 (p = 0.856)‡
2017	401 (31.3)	68 (58–78)	0.9 (0.7–1.1)	1.0 (0.7–1.4)
2018	286 (22.3)	38 (32–44)	0.5 (0.4–0.6)	0.9 (0.6–1.4)

*p values calculated by using likelihood ratio test. EU, ELISA units; GMC, geometric mean concentration; EBOV–GP, Ebola virus glycoprotein
†Adjusted for year of recruitment.
‡Adjusted for age at recruitment.

Appendix Table 3. Distribution of EBOV GP–specific binding antibody seroprevalence estimates by using different cut-offs, Sierra Leone*

Cutoff, EU/mL	No. seropositive/no. tested (%), n = 1,272	95% CI
>LLOQ (36.11)	684 (53.8)	51.0–56.5
>100	409 (32.2)	29.6–34.8
>200	274 (21.5)	19.4–23.9
>300	199 (15.6)	13.7–17.7
>400	158 (12.4)	10.7–14.4
>500	127 (10.0)	8.5–11.8
>607†	107 (8.4)	7.0–10.0

*EU, ELISA units; LLOQ, lower limit of quantification.
†Seroprevalence cutoff used for the main analysis in this study and calculated in a previous study in persons from West Africa (*8*).

Appendix Table 4. Sociodemographic characteristics and EBOV GP–specific binding antibody seropositivity among participants, Sierra Leone*

Characteristics	No. (%); n = 1,282	No. seropositive/no. tested (%)	OR (95% CI)	Adjusted OR (95% CI)†
Age group, y				
1–4	243 (19.0)	34/240 (14.2)	Referent, 1.0 (p<0.001)	1 (p<0.001)‡
5–9	170 (13.3)	61/168 (36.3)	3.5 (2.1–5.6)	3.5 (2.1–5.6)
10–19	354 (27.6)	126/353 (35.7)	3.4 (2.2–5.1)	3.4 (2.2–5.1)
20–39	390 (30.4)	145/387 (37.5)	3.6 (2.4–5.5)	3.6 (2.4–5.5)
≥40	125 (9.7)	45/124 (36.3)	3.5 (2.1–5.8)	3.4 (2.1–5.8)
Sex				
F	455 (35.5)	138/451 (30.6)	Referent, 1.0 (p = 0.332)	1 (p = 0.777)‡
M	827 (64.5)	273/821 (33.3)	1.1 (0.9–1.4)	1.0 (0.8–1.3)
Highest education level completed				
No education	362 (28.2)	82/360 (22.8)	Referent, 1.0 (p<0.001)	Referent, 1.0 (p = 0.888)
Primary, grades 1–6	378 (29.5)	135/374 (36.1)	1.9 (1.4–2.6)	0.9 (0.6–1.5)
Secondary school	480 (37.4)	170/477 (35.6)	1.9 (1.4–2.6)	0.9 (0.6–1.3)
College, university	62 (4.9)	24/61 (39.3)	2.2 (1.3–3.4)	1.0 (0.5–1.9)
Socioeconomic status of household				
Low	470 (36.7)	134/464 (28.9)	Referent, 1.0 (p = 0.104)	
Middle	396 (30.9)	130/394 (33.0)	1.2 (0.9–1.6)	
High	416 (32.5)	147/414 (35.5)	1.4 (1.0–1.8)	
No. persons in the household, adults and children, n = 1,276				
<5	274 (21.5)	83/270 (30.7)	Referent, 1.0 (p = 0.192)	
5–9	529 (41.4)	186/527 (35.3)	1.2 (0.9–1.7)	
≥10	473 (37.1)	142/469 (30.3)	1.0 (0.7–1.4)	
Number of children in the household, n = 1,274				
0–2	466 (36.6)	156/463 (33.7)	Referent, 1.0 (p = 0.725)	
3–5	536 (42.1)	166/530 (31.3)	0.9 (0.5–1.3)	
>5	272 (21.3)	87/271 (32.1)	0.9 (0.7–1.3)	
Frequency of travel out of village or town of residence, n = 1,276				
Never traveled	510 (40.0)	153/507 (30.2)	Referent, 1.0 (p = 0.252)	
Every day	19 (1.5)	10/19 (52.6)	2.6 (1.0–6.5)	
≥1×/wk	58 (4.5)	22/58 (37.9)	1.4 (0.8–2.5)	
≥1×/mo.	235 (18.4)	77/232 (33.2)	1.1 (0.8–1.6)	
<1×/mo.	454 (35.6)	148/450 (32.9)	1.1 (0.9–1.5)	

*Seropositivity defined as >99.03 ELISA Units/mL. Alternative cutoff calculated in EBOV-naive persons from the United Kingdom. EBOV GP–specific binding antibodies were indeterminate in 10 participants. p values calculated by using likelihood ratio test. EBOV–GP, Ebola virus glycoprotein; EVD, Ebola virus disease; OR, odds ratio.
†Adjusted for age and sex.
‡Age adjusted for sex. Sex adjusted for age.

Appendix Table 5. Potential EVD exposure in community or at work during the 2014–2016 Ebola outbreak and EBOV GP–specific binding antibody seropositivity among participants, Sierra Leone*

Risk factors	No. (%), n = 1,282	No. seropositive/no. tested (%)	Odds ratio (95% CI)
Living in a village/town with Ebola cases, n = 1,281			
N	199 (15.5)	57/198 (28.8)	Referent, 1.0 (p = 0.252)
Y	1,082 (84.5)	353/1,073 (32.9)	1.2 (0.9–1.7)
Knowing someone who had Ebola			
No, don't know	1,044 (81.4)	331/1,036 (32.0)	Referent, 1.0 (p = 0.565)
Y	238 (18.6)	80/236 (33.9)	1.1 (0.8–1.5)
No. EVD cases known by participant			
0	1,044 (81.4)	331/1,036 (31.9)	Referent, 1.0 (p = 0.608)
1	125 (9.8)	39/125 (31.2)	1.0 (0.6–1.4)
2–3	66 (5.2)	26/65 (40.0)	1.4 (0.9–2.4)
>3	47 (3.7)	15/46 (32.6)	1.0 (0.5–1.9)
Closest relationship with an EVD case, n = 1,280			
No relationship†	1,044 (81.5)	331/1,036 (32.0)	Referent, 1.0 (p = 0.500)
Close family‡	27 (2.1)	7/27 (25.9)	0.7 (0.3–1.8)
Other relative	52 (4.1)	16/51 (31.4)	1.0 (0.5–1.8)
Friend	59 (4.6)	18/59 (30.5)	0.9 (0.5–1.7)
Community member	98 (7.7)	39/97 (40.2)	1.4 (0.9–2.2)
Living in the same household with an EVD case, n = 1,280			
N	1,269 (99.1)	407/1,260 (32.3)	Referent, 1.0 (p = 0.876)
Y	11 (0.9)	3/10 (30.0)	0.9 (0.2–3.5)
Caring for an EVD case, n = 1,281			
N	1,272 (99.3)	408/1,262 (32.3)	Referent, 1.0 (p = 0.504)

Risk factors	No. (%), n = 1,282	No. seropositive/no. tested (%)	Odds ratio (95% CI)
Y	9 (0.7)	2/9 (22.2)	0.6 (0.1–2.9)
Direct body contact with an EVD case, n = 1,281			
N	1,275 (99.5)	408/1,265 (32.3)	Referent, 1.0 (p = 0.955)
Y	6 (0.5)	2/6 (33.3)	1.1 (0.2–5.8)
Attending a funeral of an EVD case			
N	1,263 (98.5)	404/1,254 (32.2)	Referent, 1.0 (p = 0.554)
Y	19 (1.5)	7/18 (38.9)	1.3 (0.5–3.5)
Health care frontline worker during EVD outbreak			
No, NA§	1,254 (97.8)	403/1,244 (32.4)	Referent, 1.0 (p = 0.665)
Y	28 (2.2)	8/28 (28.6)	0.8 (0.4–1.9)

*Seropositivity defined as >99.03 ELISA units/mL. Alternative cutoff calculated in EBOV-naive persons from the United Kingdom. EBOV GP–specific binding antibodies were indeterminate in 10 participants. p values calculated by using likelihood ratio test. Because none of the variables was associated with seropositivity in univariable analysis, the adjusted odds ratio column is omitted from the table. EBOV GP, Ebola virus glycoprotein; EVD, Ebola virus disease; NA, not applicable.
†No relationship; participant did not know anyone with Ebola.
‡Participant was the parent or child or spouse or sibling of an EVD case.
§Not applicable; participant was a child or did not have a job.

Appendix Table 6. Potential risk factors for transmission of Ebola virus from animals during the 2014–2016 Ebola outbreak and EBOV GP–specific binding antibody seropositivity among participants, Sierra Leone*

Risk Factors	No. (%), n = 1,282	No. seropositive/no. tested (%)	OR (95% CI)	Adjusted OR (95% CI)†
Number of domestic animals in the participant’s compound				
0	503 (39.2)	150/498 (30.1)	Referent, 1.0 (p = 0.362)	
1–5	374 (29.2)	122/371 (32.8)	1.1 (0.9–1.5)	
>5	405 (31.6)	139/403 (34.5)	1.2 (0.9–1.6)	
Having the following domestic animals in the compound, n = 1,281‡				
Dog				
N	1,116 (87.1)	353/1,107 (31.9)	Referent, 1.0 (p = 0.377)	
Y	165 (12.9)	58/164 (35.4)	1.2 (0.8–1.6)	
Cat				
N	951 (74.2)	304/943 (32.2)	Referent, 1.0 (p = 0.898)	
Y	330 (25.8)	107/328 (32.6)	1.0 (0.8–1.3)	
Goat, sheep				
N	870 (67.9)	277/863 (32.1)	Referent, 1.0 (p = 0.790)	
Y	411 (32.1)	134/408 (32.8)	1.0 (0.8–1.3)	
Pig				
N	1,263 (98.6)	399/1,253 (31.8)	Referent, 1.0 (p = 0.003)	Referent, 1.0 (p = 0.004)
Y	18 (1.4)	12/18 (66.7)	4.3 (1.6–11.5)	4.1 (1.5–11.4)
Other				
N	825 (64.4)	258/817 (31.6)	Referent, 1.0 (p = 0.439)	
Y	456 (35.6)	153/454 (33.7)	1.1 (0.9–1.4)	
Touching sick or dead domestic animals				
N	1,253 (97.7)	400/1,243 (32.2)	Referent, 1.0 (p = 0.518)	
Y	29 (2.3)	11/29 (37.9)	1.3 (0.6–2.8)	
Hunting for wild animals§				
N	1,261 (99.3)	404/1,251 (32.3)	Referent, 1.0 (p = 0.947)	
Y	9 (0.7)	3/9 (33.3)	1.0 (0.3–4.2)	
Touching sick or dead wild animals				
N	1,277 (99.6)	410/1,267 (32.4)	Referent, 1.0 (p = 0.538)	
Y	5 (0.4)	1/5 (20.0)	0.5 (0.1–4.7)	
Consumption of bush meat				
N	1,275 (99.4)	409/1,265 (32.3)	Referent, 1.0 (p = 0.830)	
Y	7 (0.6)	2/7(28.6)	0.8 (0.2–4.3)	

*Seropositivity defined as >99.03 ELISA units/mL. Alternative cutoff calculated in EBOV-naive persons from the United Kingdom. EBOV GP–specific binding antibodies were indeterminate in 10 participants. p values calculated by using likelihood ratio test. EBOV–GP, Ebola virus glycoprotein.
†Adjusted for age and sex.
‡Participants could indicate >1 type of domestic animal.
§Types of wild animals hunted by participants who answered yes included monkeys, duiker antelopes, bats, and rodents.

Appendix Table 7. Additional sociodemographic characteristics of the study population not included in the risk factor analysis of Ebola virus IgG seroprevalence, Sierra Leone

Characteristics	No. (%); n = 1,282
Occupation	
Salaried employment	74 (5.8)
Self-employed, e.g., trader or farmer	211 (16.5)
Housewife	18 (1.4)
Unemployed	78 (6.1)
Student or apprentice	635 (49.5)
Preschool child	259 (20.2)
Other	7 (0.5)
Religion*	
Muslim	1,062 (82.9)
Christian	217 (16.9)
None	2 (0.2)
Tribe	
Themne	861 (67.2)
Limba	159 (12.4)
Soso	115 (9.0)
Fula	36 (2.8)
Mende	44 (3.4)
Other	67 (5.2)

*Religion not available for 1 participant.

Appendix Table 8. Additional travel information for persons reporting travel outside their village or city of residence during the Ebola virus disease outbreak, Sierra Leone, March 2014–January 2016

Characteristics	No. (%); n = 770*
Destination of most recent journey†	
Major cities, i.e., Freetown	361 (46.9)
Village in the same chiefdom	172 (22.3)
Different chiefdom within same district	136 (17.7)
Another district within Sierra Leone	43 (5.6)
Guinea	49 (6.4)
Traveling time to the farthest destination‡	
<1 h	148 (19.4)
1–2 h	251 (32.9)
3–6 h	344 (45.2)
All day, >1 d	19 (2.5)
Purpose of the trip	
Visiting someone	498 (64.7)
Work, business	141 (18.3)
Attending a funeral	22 (2.8)
Attending another event§	36 (4.7)
Seeking healthcare	9 (1.2)
Accompanying somebody	13 (1.7)
Study or holiday	16 (2.1)
Other reasons	35 (4.5)

*N = 770 correspond to 766 participants who reported a travel frequency in Appendix Table 1 plus 4 participants with missing data on travel frequency but who reported a travel destination for their most recent journey outside their village/town of residence.
†Participants could indicate more than one destination; information not available for 40 participants.
‡Information not available for 8 participants.
§Other events included weddings, feasts, football matches, and religious ceremonies.

Appendix Table 9. Information on illness or medical issues during the Ebola virus disease outbreak, Sierra Leone, March 2014–January 2016*

Characteristics	No. (%); n = 1,282
Being unwell during the EVD outbreak	
Y	219 (17.1)
N	1,051 (82.0)
Don’t know, don’t remember	11 (0.9)
Participants who reported being unwell during the EVD outbreak, n = 219†	
Medical issues or symptoms	
Headache	169 (77.2)
Fever	111 (50.7)
Vomiting	25 (11.4)
Diarrhea	18 (8.2)
Joint and muscle pain	73 (33.3)
Rash	17 (7.8)
Muscle weakness	39 (17.8)
Other symptoms	30 (13.7)
Duration of symptoms	
Few hours	51 (23.3)
1–2 d	96 (43.8)
About 1 week	47 (21.5)
>1 week	22 (10.0)
Don’t know	3 (1.4)
Seen by a doctor or nurse, n = 216	
Y	97 (44.9)
N	119 (55.1)
Any condition diagnosed, n = 216	
Y‡	80 (37.0)
N	11 (5.1)
Don’t know, don’t remember	6 (2.8)
Not applicable§	119 (55.1)
Given any treatment, n = 216	
Y	94 (43.5)
N	2 (0.9)
Don’t know, don’t remember	1 (0.5)
Not applicable§	119 (55.1)
Female participants of childbearing potential, aged 16–50 y, n = 157	
Experienced a miscarriage during the EVD outbreak	
Y	2 (1.6)
N	125 (98.4)
Experienced a stillbirth during the EVD outbreak	
Y	1 (0.8)
N	126 (99.2)

*EVD, Ebola virus disease.
†Percentages calculated only among the participant who reported being unwell during the EVD outbreak, n = 219. Information not available in 3 participants.
‡Diagnoses: malaria (n = 45); typhoid fever/diarrhea with or without concomitant malaria infection (n = 9); pneumonia (n = 1); pulmonary tuberculosis (n = 1); other conditions (n = 14); no diagnosis available (n = 14).
§Not applicable participants were not seen by a doctor.
